# Interrelations between insomnia, dreaming, and schizotypy in the general population: A network model

**DOI:** 10.1192/j.eurpsy.2022.902

**Published:** 2022-09-01

**Authors:** N. Báthori, B. Polner, P. Simor

**Affiliations:** 1 Budapest University of Tehcnology and Economics, Department Of Cognitive Science, Budapest, Hungary; 2 Budapest University of Technology and Economics, Department Of Cognitive Science, Budapest, Hungary; 3 Eötvös Loránd University, Institute Of Psychology, Budapest, Hungary

**Keywords:** dreaming, network model, schizotypy, Insomnia

## Abstract

**Introduction:**

Insomnia and Nightmare disorder are the two most common comorbid sleep disturbances in psychotic conditions. However, insomnia and psychotic symptoms are umbrella terms that hide the heterogeneity of these concepts. Several studies have found that worsening sleep quality is associated with the strengthening of psychotic symptoms. Until now, there was less interest in the relationship between the specific insomnia symptoms (trouble with falling asleep, fragmented sleep, early awakenings, daytime consequences) and the specific dimensions of schizotypy (disorganization, unusual perceptual experiences, anhedonia, and impulsive nonconformity).

**Objectives:**

The study aimed to depict the network structure of insomnia, dreaming features (dream recall/bad dream/nightmare frequency), and schizotypy dimensions.

**Methods:**

Exploratory network analysis was conducted on cross-sectional data of the general population (N=1419, 77 % female). We modeled the interrelations between insomnia symptoms (Athens Insomnia Scale), dreaming features (the frequency of dream recall/bad dreams/nightmares), and the dimensions of schizotypy (OLIFE-S).

**Results:**

show a highly connected network with strong stability. The nodes of schizotypy, insomnia, and dream feature perfectly correspond to their own clusters, but the nodes were also densely connected between the three clusters. Disorganization, frequent awakenings, and nightmares are the most central nodes of the clusters. The node of frequent nightmares seems to be the bridge symptom in this network which connects unusual experiences dimension and frequent awakenings.

**Conclusions:**

These results suggest that specific dimensions of schizotypy and specific sleep complaints are differently connected. However further research is needed to investigate the finer details of these heterogenic phenomena.

**Disclosure:**

No significant relationships.

